# Modulation of Intestinal Epithelial Permeability by Plasma from Patients with Crohn’s Disease in a Three-dimensional Cell Culture Model

**DOI:** 10.1038/s41598-018-38322-8

**Published:** 2019-02-14

**Authors:** Pan Xu, Elhaseen Elamin, Montserrat Elizalde, Paul P. H. A. Bours, Marieke J. Pierik, Ad A. M. Masclee, Daisy M. A. E. Jonkers

**Affiliations:** 1 Division of Gastroenterology-Hepatology, Department of Internal Medicine, Maastricht University Medical Centre, Maastricht, The Netherlands; 2 School of Nutrition and Translational Research in Metabolism, Maastricht University Medical Center, Maastricht, The Netherlands

## Abstract

Intestinal epithelial barrier is affected by multiple factors, such as tumour necrosis factor-α (TNF-α). Plasma concentration of TNF-α is higher in patients with Crohn’s disease (CD) than healthy controls (HC) and correlates positively with disease activity. This study aimed to determine the effect of plasma from active, inactive CD patients on intestinal barrier function and to investigate the underlying mechanism. Plasma samples were collected from CD patients and HC. 3D Caco-2 cysts were treated with plasma or TNF-α, with or without pre-incubation of adalimumab (a monoclonal antibody that antagonizes TNF-α) or JNK inhibitor SP600125. The results demonstrated that exposure of the cysts to plasma from CD patients resulted in enhanced paracellular permeability in a disease activity-dependent manner. Compared to HC, active CD plasma decreased ZO-1 and OCCLUDIN expression on mRNA and protein levels, and led to an increased JNK phosphorylation. Pre-incubation with adalimumab or SP600125 ameliorated TJ disruption and barrier dysfunction induced by plasma from CD patients. These results indicate that plasma from CD patients is able to induce epithelial barrier disruption, in part through TNF-α induced TJs modulation. The data also demonstrate an involvement of MAPK pathway, in particular the JNK isoform, in CD patient plasma-induced barrier dysfunction.

## Introduction

Inflammatory bowel disease (IBD), comprising ulcerative colitis (UC) and Crohn’s disease (CD), is characterized by chronic relapsing intestinal inflammation that leads to debilitating (extra-) intestinal complications and a reduced quality of life in most patients^1^. Active CD is characterised by mucosal inflammation which is typically patchy, occurring throughout the gastrointestinal tract and can be transmural^2^. Aadequate treatment of active disease is important to improve long term outcome and prevent complications to occur. Inactive disease is generally referred to as remission. The pathogenesis of CD is complex and still has not been fully elucidated. However, it is thought to involve a tangle interplay among environmental, immunological and microbial factors in genetically susceptible hosts^2^. Among others, pro-inflammatory cytokines have been implicated in the pathogenesis of IBD, where they appear to have a central role in regulating intestinal inflammation. Mucosal as well as systemic concentrations of several cytokines including tumour necrosis factor-α (TNF-α), interferon-γ (IFN-γ), interleukin-1β (IL-1β) were found to be markedly increased in patients with CD when compared to healthy control subjects and correlated positively with disease activity^3–5^. Moreover, recent advances have highlighted a crucial role of impaired epithelial integrity in disease pathophysiology^6,7^. A defective mucosal barrier may result in increased permeation of luminal contents, triggering an immune response that stimulates and/or accelerates mucosal inflammation^2^. Indeed, a significant correlation has been established between altered intestinal permeability and disease activity in CD patients^7–11^. Earlier clinical studies also documented that changes in intestinal permeability could predict CD disease course^6,12,13^, while some even define IBD as an impaired intestinal barrier disease^14^.

The intestinal epithelium provides a selectively permeable barrier, permitting absorption of luminal water and nutrients while limiting influx of noxious substances, including microorganisms and their products, into the systemic circulation and bowel wall^15^. The intestinal barrier is maintained in a large part by intercellular junctional proteins consisting of tight junctions (TJ) and adherens junctions (AJ)^16^. The TJ are composed of multiple proteins including the transmembrane proteins occludin, the claudin family, junctional adhesion molecule (JAM), the cytoplasmic proteins zona occludens-1, -2 and -3 (ZO-1, -2, -3)^16^, and tricellular tricellulin and angulins^17^. The AJ consist of the transmembrane protein E-cadherin that interacts with the cytoplasmic protein β-catenin^15^. Alterations in distribution and expression of TJ and AJ have been shown in inflamed mucosa of CD patients^16,18–21^.

Intestinal barrier integrity is regulated by multiple factors including nutrients, commensal gut bacteria, cytokines and immune cells. Notably, despite the fact that many of those factors such as lipopolysachariden (LPS), TNF-α, and IL-17 (+) immune cells were found to be increased in blood of CD patients compared to healthy subjects^22^, it is yet not known whether the systemic circulation from CD patients, as a whole compartment, confers a substantial effect on intestinal barrier. In particular, TNF-α as a central pro-inflammatory mediator in CD, has been shown to impair TJ expression or localization and subsequently induces barrier dysfunction^23–25^. *In vitro* studies using intestinal epithelial monolayers revealed that TNF-α induces barrier dysfunction through a mechanism that is primarily mediated by myosin light chain kinase (MLCK) activation^26^. This notion is further supported by *in vivo* studies demonstrating an improved intestinal permeability in patients responding to anti-TNF therapy^27,28^. In addition to the TNF-α-MLCK cascade, the mitogen-activated protein kinase (MAPK) transduction pathway has also been found to be implicated in CD disease course^29^. Sustained activation of the extracellular signal-regulated kinases (ERK) 1/2, the p38 kinases and the c-Jun N-terminal kinases (JNKs) has been observed in the inflamed mucosa of CD patients^29^. However, the majority of previous research on the role of MAPK in CD has focused on its involvement in the inflammatory responses and cross-talk to other inflammatory pathways, such as NF-kB and Janus kinase/STAT signalling^29^. Their function in mediating intestinal barrier defects has not been fully elucidated. In particularly, recent advances have demonstrated JNK pathway as a potential target for IBD therapy, the beneficial effects of JNK inhibitors in reducing intestinal inflammation are currently under exclusive investigations, whether the JNK isoform and its inhibitors converge intestinal barrier function to regulate pathological process in CD is largely unknown. Therefore, in this study, our first aim was to determine the effects of plasma from active, inactive CD patients and healthy controls on paracellular permeability and TJ integrity. Second, we investigated the role of the MAPK as potential underlying signalling mechanism involved in CD plasma-modulated barrier function. For the current study, a three-dimensional (3D) Caco-2 cell culture model was employed, which more accurately recreates the features of intestinal epithelium *in vivo* and offers a great promise to examine the responsiveness and underlying mechanisms of epithelial cells to pathophysiological stimuli.

## Results

### Effects of plasma from CD patients on paracellular permeability

**Figure 1:**
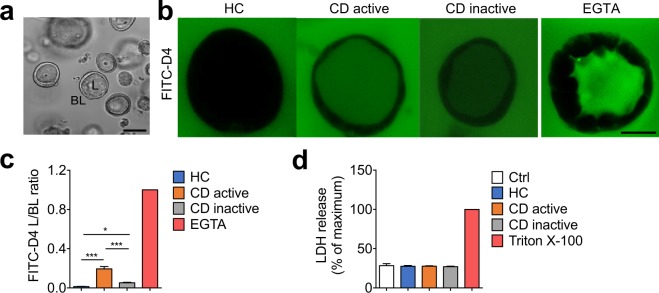
Effects of plasma from patients with Crohn’s disease (CD) on paracellular permeability in 3D Caco-2 cysts. (**a**) Representative Caco-2 cysts with luminal and basal compartments indicated. The bar indicates 100 µm (**b**) Representative images showing the intraluminal accumulation of FITC-dextran-4 (FITC-D4, green) in the cysts treated with plasma (37.5% v/v) from healthy controls (HC), active or inactive CD patients, or EGTA (2 mM, positive control) for 24 hours. Images were captured from the middle of the cysts using confocal microscopy. The bar indicates 50 µm. (**c**) The mean fluorescence intensity of FITC-D4, expressed as the ratio of the luminal (L) over the basal (BL) compartment, after the abovementioned plasma exposure. The L/BL ratio of EGTA exposure was set to 1. (**d**) Effect of the abovementioned plasma treatments on LDH release in Caco-2 cysts. Triton X-100 was used as positive control to induce maximum LDH leakage. Data are reported as percentage of maximum LDH release. All graphs are expressed as means ± SEM of three independent experiments with 6 subjects per group and at least 8 cysts per subject. *P < 0.05; **P < 0.01; ***P < 0.001 by one-way ANOVA and Tukey’s post-hoc test.

Intestinal epithelial Caco-2 cells were cultured to form hollow 3D cysts with a single layer of cells following a previously described protocol^30^ (Fig. 1a), and then exposed basolaterally to plasma from HC, active and inactive CD patients. EGTA, which is known to effectively impair barrier function, was used as a positive control. Upon HC plasma treatment, FITC-D4 was exclusively observed at the basolateral side of cysts, resulting in a very low L/BL fluorescence ratio, while exposure to active and inactive CD plasma dramatically increased intraluminal FITC-D4 signals, as evidenced by a significant increase of the L/BL fluorescence ratio (Fig. 1b,c, 0.194 ± 0.024 and 0.052 ± 0.005 vs. 0.012 ± 0.002, *P* < 0.001 and *P* < 0.05 respectively). Interestingly, a significant reduction of FITC-D4 permeation in the lumen was observed when cysts were exposed to plasma from inactive compared to the active CD patients (Fig. 1b,c, *P* < 0.001). Similar effects were also observed using conventional 2D Caco-2 culture model. We observed that exposing Caco-2 monolayers to plasma from CD patients resulted in a significant, though less pronounced reduction of the TEER values in a disease activity-dependent manner (Supplementary Fig. 1).

To investigate whether the increased permeation of FITC-D4 was due to cell lysis, effects of the plasma treatment (37% v/v) on 3D Caco-2 cell viability were determined by measuring LDH release. No significant reduction in LDH activity was detected for any of the groups when compared to maximum LDH leakage induced by Triton X-100 (Fig. 1d).

### Effects of plasma from CD patients on tight junction integrity

**Figure 2:**
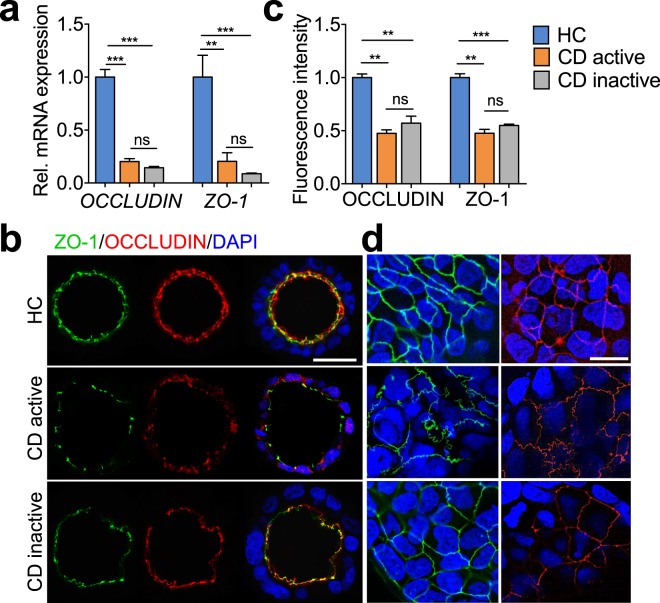
Effects of plasma from patients with Crohn’s disease (CD) on OCCLUDIN and ZO-1 in 3D Caco-2 cysts. mRNA levels (**a**), confocal microscopy analyses of OCCLUDIN and ZO-1 (**b–d**) in Caco-2 cysts that were treated with plasma (37.5% v/v) from healthy controls (HC), active or inactive CD patients for 24 hours. Images in (**b**) were captured from the middle cross-section of the cysts. Bar indicates 50 µm. Quantified fluorescence intensities in (**b**) are shown in (**c**). Images in (**d**) were captured from the top surface of the cysts. Bar indicates 20 µm. All graphs represent the results of three replicate experiments. Data expressed as means ± SEM with 6 subjects per group and at least 8 cysts per subject. **P < 0.01; ***P < 0.001 by one-way ANOVA and Tukey’s post-hoc test.

The epithelial paracellular permeability is largely regulated by intercellular junctional complex. To understand the modulating effect of patient-derived plasma on paracellular permeability, we assessed the gene expression of key cell junction proteins, such as *OCCLUDIN*, *ZO-1, TRICELLULIN, LSR, ILDR-1* and *ILDR-2*. In parallel to the observed changes in FITC-D4 permeability (Fig. 1b,c), exposure to active and inactive CD plasma (37.5% v/v) significantly blunted the mRNA levels of *OCCLUDIN*, *ZO-1* as compared to HC plasma (Fig. 2a, 0.203 ± 0.027 and 0.144 ± 0.013 vs. 1 ± 0.073, *P* < 0.001; 0.205 ± 0.081 and 0.088 ± 0.007 vs. 1 ± 0.207, *P* < 0.001). However, no significant difference was observed under inactive CD plasma exposure as compared to the active (Fig. 2a). The mRNA expression profiles of *ZO-1* and *OCCLUDIN* were accompanied by alterations of their protein levels, as evidenced by immunofluorescent staining of both molecules. Incubation of both active and inactive CD plasma resulted in a profound loss of ZO-1 and OCCLUDIN at the intercellular junctions when compared to HC plasma-treated cysts (Fig. 2b,c), as well as deformed sinuous TJ belts at bicellular contacts (Fig. 2d). No significant distortion from apical-side towards basal-side was observed in any of the treatments (Fig. 2d). In addition, despite no significant difference in protein levels of ZO-1 and OCCLUDIN between active and inactive CD plasma treatments, exposure of active CD plasma yielded a more pronounced sinuous junction morphology compared to inactive CD plasma (Fig. 2d). Additionally, qRT-PCR analyses demonstrated undetectable expressing levels of *LSR, ILDR-1, ILDR-2* in Caco-2 cells. No significant changes of *TRICELLULIN* were found among treatments of plasma from active, inactive or HC (Supplementary Fig. 2).

### Involvement of TNF-α in CD plasma-mediated barrier function

**Figure 3:**
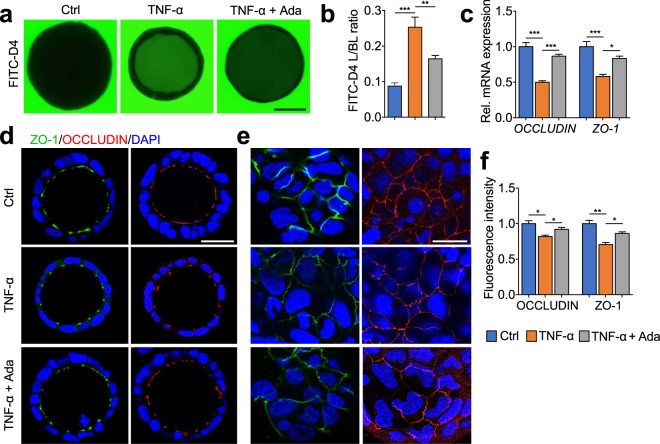
Effects of TNF-α and adalimumab on paracellular permeability, OCCLUDIN and ZO-1 in 3D Caco-2 cysts. Caco-2 cysts were exposed at the basal side to medium only (Ctrl), TNF-α (25 pg/mL) with or without pre-incubation of adalimumab (20 μg/mL) for 24 hours. (**a**) Representative images showing the intraluminal accumulation of FITC-dextran-4 (FITC-D4, green) in the Caco-2 cysts. Bar indicates 50 μm. (**b**) The mean FITC-D4 intensity of Caco-2 cysts measured and expressed as the L/BL ratio of the luminal (L) over the basal (BL) compartment. (**c**) mRNA levels of *OCCLUDIN* and *ZO-1* in 3D Caco-2 cysts. (**d–f**) Confocal microscopy analyses of ZO-1 (green), OCCLUDIN (red) and nuclei (blue). Images in (**d**) were captured from the middle cross-section of 3D cysts. Bar indicates 50 μm. Images in (**e**) were captured from the top surface of 3D cysts. Bar indicates 20 μm. Quantified fluorescence intensities in (**d**) are shown in (**f**). All graphs represent the results of three replicate experiments. Data expressed as means ± SEM with at least 8 cysts per treatment. *P < 0.05; **P < 0.01; ***P < 0.001 by one-way ANOVA and Tukey’s post-hoc test.

As TNF-α, a key pro-inflammatory cytokine in CD, has been shown to impair TJ integrity and epithelial barrier function^24^, we hypothesized that the impact of plasma from CD patients on barrier function is attributed to TNF-α. Incubation of Caco-2 cysts with TNF-α (25 pg/mL) increased barrier permeability significantly (Fig. 3a,b, 0.253 ± 0.028 vs. 0.087 ± 0.009, *P* < 0.001), which was ameliorated by pre-incubation of cysts with 20 μg/mL adalimumab, a concentration commonly found in sera from treated patients lacking anti-adalimumab antibodies^31^ (Fig. 3a,b, 0.164 ± 0.009 vs. 0.253 ± 0.028, *P* < 0.01). In parallel, exposure to TNF-α to 3D Caco-2 cyst also attenuated the TJs integrity via dysregulating ZO-1 and OCCLUDIN on both mRNA and protein levels, and inducing deformed sinuous TJ belts at bicellular contacts (Fig. 3c–f). These effects were further ameliorated by the supplementation of adalimumab (Fig. 3c–f). In support of this, similar effects were observed in 2D Caco-2 cell culture model. Treatment of Caco-2 monolayers to TNF-α (10 ng/mL) led to a significantly decrease of TEER values as compared to control, which was prevented by pre-incubation of adalimumab (Supplementary Fig. 3a,b).

**Figure 4:**
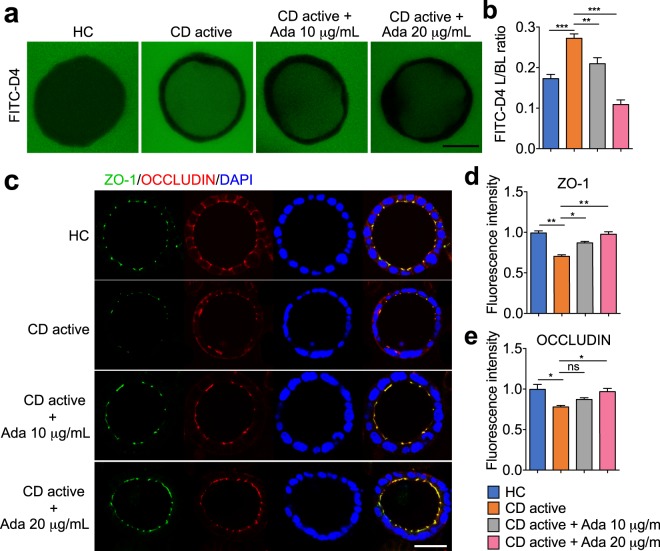
Pre-treatment of adalimumab attenuates the effects of plasma from Crohn’s disease (CD) patients on paracellular permeability, OCCLUDIN and ZO-1 expression of 3D Caco-2 cysts. Cysts were exposed at the basal side to plasma (37.5% v/v) from healthy controls (HC) or active CD patients with or without pre-incubation of adalimumab (10 or 20 μg/mL) for 24 hours. (**a**) Representative images showing the intraluminal accumulation of FITC-D4 (green) in Caco-2 cysts. Bar indicates 50 μm. (**b**) The mean fluorescence intensity of FITC-D4 measured and expressed as the L/BL ratio of the luminal (L) over the basal (BL) compartment. Representative images (**c**), and fluorescence intensities (**d–e**) from confocal microscopy analyses of ZO-1, OCCLUDIN in 3D Caco-2 cysts, Images were captured from the middle cross-section of the cysts. Bar indicates 50 μm. All graphs represent the results of three replicate experiments. Data expressed as means ± SEM with at least 8 cysts per subject (6 subjects per group) or per treatment. *P < 0.05; **P < 0.01; ***P < 0.001 by one-way ANOVA and Tukey’s post-hoc test.

To further investigate the importance of TNF-α as a mediator in the plasma-modulated epithelial permeability, Caco-2 cysts were pre-treated with 10 or 20 μg/mL adalimumab and then incubated with plasma. We observed that pre-incubation of adalimumab significantly reduced the FITC-D4 flux and L/BL fluorescence ratio in a dose dependent manner compared to CD plasma alone (Fig. 4a,b, 0.210 ± 0.015 and 0.109 ± 0.012 vs. 0.272 ± 0.011, *P* < 0.01 and *P* < 0.001 respectively). Consistent with this, pre-treatment of adalimumab (20 μg/mL) in 2D Caco-2 monolayers also prevented the TEER drop induced by active CD plasma (Supplementary Fig. 1). In parallel, while plasma from active CD patients induced a reduction in ZO-1 and OCCLUDIN proteins, pre-administration of adalimumab (20 μg/mL) substantially restored their abundance (Fig. 4c–e).

### Involvement of MAPK pathway in CD plasma-mediated barrier function

**Figure 5:**
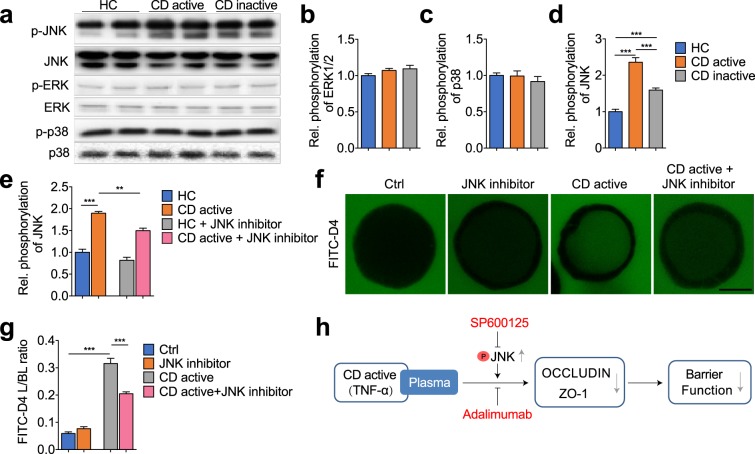
MAPK signalling mediates CD plasma induced disruption of barrier function in 3D Caco-2 cysts. (**a–e**) Phosphorylation levels of ERK1/2, p38 and JNK in Caco-2 cysts that were exposed to plasma from healthy control (HC), active or inactive CD patients, with or without pre-treatment of JNK inhibitor SP600125 (100 nmol/L). (**f**,**g**) Representative images showing the intraluminal accumulation of FITC-D4 (green) in Caco-2 cysts (**f**), and the mean fluorescence intensity of FITC-D4 measured and expressed as the L/BL ratio of the luminal (L) over the basal (BL) compartment (**g**) in 3D Caco-2 cysts that were exposed to plasma from healthy control (HC) or active CD, with or without pre-treatment of JNK inhibitor SP600125. Bar indicates 50 μm. (**h**) Graphical summary illustrating how plasma from CD active patients promotes barrier dysfunction. Data are expressed as means ± SEM of at least 8 cysts per condition, with 6 subjects per group. **P < 0.01; ***P < 0.001 by one-way ANOVA and Tukey’s post-hoc test.

We further elucidated the involvement of the MAPK signalling pathway in plasma-modulated barrier function. Phosphorylation levels of ERK1/2, p38 and JNK were assessed using cell-based ELISA and western blotting. As shown in Fig. 5a–d, incubation with active and inactive CD plasma led to no significant changes of ERK1/2 and p38 phosphorylation when compared with HC plasma treatment. Notably, we observed a significant activation of JNK phosphorylation when the cysts were exposed to active or inactive CD plasma (Fig. 5a,d, 2.360 ± 0.127 and 1.593 ± 0.060 vs. 1 ± 0.069, *P* < 0.001). Increased p-JNK to total JNK was further prevented by pre-treatment with SP600125 (100 nmol/L), a potent JNK inhibitor (Fig. 5e, 1.495 ± 0.060 vs. 1.896 ± 0.039, *P* < 0.01). To further delineate the role of JNK, we evaluated the effects of SP600125 on CD plasma-induced barrier dysfunction. We observed that pre-treatment of both Caco-2 3D cysts and 2D monolayers with SP600125 showed a significant decline in FITC-D4 permeation (Fig. 5f,g, 0.205 ± 0.007 vs. 0.316 ± 0.019. *P* < 0.001) and TEER values (Supplementary Fig. 1) respectively, in response to active CD plasma treatment, which suggested a distinct role for the JNK isoform in the mediation of CD plasma-induced barrier dysfunction.

## Discussion

In the present study, using a 3D Caco-2 cell culture model, we evaluated the effects of plasma from patients with Crohn’s disease (CD) on epithelial barrier function. We demonstrated that plasma from active and inactive CD patients, compared to healthy controls, triggers a disruption of the epithelial tight junctions, which results in increased paracellular permeability, while the effect of plasma from inactive CD patients was less pronounced as compared to the active. This was at least in part mediated by TNF-α, involving activation of the MAPK signalling pathway, in particularly through the JNK isoform. Pre-incubation with the anti-TNF biological adalimumab or the JNK inhibitor both ameliorated the barrier disruption induced by plasma from CD patients (Fig. 5h).

Epithelial barrier dysfunction is increasingly recognized as a hallmark that contributes to the pathogenesis of CD^32^. However, studies addressing the role of systemic compartment and key triggers of an increased intestinal permeability are so far very limited. Previous investigations revealed different cytokine profiles in plasma of CD patients compared to healthy controls, including increased TNF-α levels^4,5,33^. Herein, using a well-established Caco-2 3D culture model, we examined the interaction between plasma from CD patients and intestinal epithelial cells. Our data showed that exposure of the cysts to plasma from CD patients results in enhanced paracellular permeability in a disease activity-dependent manner, without compromising cell viability.

As disruption of the barrier junctional proteins is known to contribute to changes in epithelial permeability, expression of key bicellular and tricellular TJ components were examined. Our data showed that exposure to CD plasma did decrease ZO-1 and OCCLUDIN expression on both mRNA and protein levels, coupled with deformed TJ belts, indicating that the observed changes in paracellular permeability were attributable to disruption of TJ integrity. Furthermore, exposure of Caco-2 cysts to plasma from active CD patients induced a more leaky barrier compared to inactive CD patients. ZO-1 and OCCLUDIN, however, did not show significant differences in protein contents or localization between active and inactive CD plasma treatments. In contrast, a more pronounced sinuous junction morphology was noted upon the exposure of active CD plasma as compared to inactive CD (Fig. 2d), which may contribute to the more disrupting effect of active CD plasma. Collectively, these findings reveal that plasma compartment is a key factor that regulates barrier function, which could contribute to CD disease progression.

The pro-inflammatory cytokine TNF-α has been extensively characterized as a key player in the pathogenesis of CD^34,35^. Normally, TNF-α is hardly detectable in healthy individuals, but elevated plasma levels are found in inflammatory and infectious conditions^3–5^. In the present study, the HC did not suffer from any inflammatory GI or other chronic disorders, nor from an infection at the time of inclusion. The secretion patterns of TNF-α from patients with CD demonstrated a positive correlation with disease activity and extent of inflammation^3–5,36^. To better understand the potential mechanisms underlying the plasma-induced epithelial barrier integrity in CD, we examined whether the effects can be, at least in part, attributed to TNF-α. Caco-2 cysts incubated with 25 pg/mL TNF-α for 24 hours displayed a significant increase of cell permeability, reduction of ZO-1 and OCCLUDIN protein contents, and deformed TJ belts, which were prevented by pre-administration with adalimumab. Adalimumab is a recombinant human immunoglobulin-G1 monoclonal antibody that binds to TNF-α and inhibits its interaction with TNF-α receptors. By blocking TNF-α, adalimumab reduces abnormal and progressive inflammatory processes. Treatment of adalimumab to CD patients was found to induce mucosal healing, fistula closure, and is proven to be effective for induction and maintenance of deep remission^37^. Based on these landmark studies, adalimumab therapy has been approved for use in patients with moderate-to-severe CD who are refractory to conventional therapy^27,38^. In our experimental settings, both TNF-α (25 pg/mL) and adalimumab (10 and 20 μg/mL) were applied at a physiological range of concentrations^39^ without showing any significant cell cytotoxicity (data not shown). These results are akin to previous *in vitro* studies using Caco-2, T84, or HT-29/B6 monolayers^23,26,40,41^, although the precise effects largely depend on the cell type. Moreover, pre-incubation with adalimumab effectively prevented the barrier disruption induced by active CD plasma, and the blockage was almost complete when adalimumab was applied at 20 μg/mL, confirming that TNF-α was the major factor responsible for the observed effect of the plasma. While previous studies on anti-TNF therapy have focused primarily on inflammation, our current results demonstrated a clear potential of the anti-TNF drug on epithelial barrier restoration. This notion is also supported by findings obtained with another anti-TNF-α antibody, infliximab, demonstrating its ability to improve barrier function in patients suffering from CD^28,42^ and to antagonize structural changes of the TJs in mice with colitis^43^. Our data therefore support the concept that strengthening the intestinal barrier is a potential mechanism that contributes to the clinical efficacy of adalimumab in CD patients. On the other hand, it is worth mentioning that, in addition to TNF-α, there might be other factors attributable for the more impaired barrier function of active CD plasma as compared to the inactive, including other pro-inflammatory cytokines, bacterial outer-membrane vesicles^44^ or metabolites^45^, which remain to be investigated in further studies.

Molecular events underlying the regulation of intestinal permeability in IBD have been the subject of intense investigation over the past years, as this can provide leads for preventive strategies and improved therapeutic. Strong evidence has revealed a crucial role for MAPK signalling pathway in the pathogenesis of IBD. Different isoforms of MAPK pathway p38, ERK1/2 and JNK were shown to be significantly activated in the inflamed intestinal mucosa of IBD patients^29^. Further investigations demonstrated that MAPK cascades share extensive networks with inflammatory pathways. Among these, TNF-α is one of the best-characterized agonists of the p38 and JNK pathways and itself is regulated by p38 and JNK^29^. Other cytokines, such as IL-16, which is up-regulated in CD also activates JNK and p38^46^. Despite the in-depth characterization of MAPK signalling in inflammation, its potential linkage to epithelial barrier function is still largely unexplored. In this study, exposure of the Caco-2 cysts to the plasma from active CD patients led to increased phosphorylation of JNK, but did not affect ERK1/2 and p38 phosphorylation. Concurrent administration of a JNK inhibitor could ameliorate barrier disruption induced by plasma from active CD patients, assigning a crucial role for JNK signalling in the regulation of barrier function. This is of particular interest, because previous investigations have mainly focused on the involvement of ERK1/2^47–49^ and p38^50,51^ in the regulation of epithelial barrier function. It has been demonstrated that activation of p44/42 ERK led to an increased expression of claudin-4 in MDCK cells^47^. In Caco-2 cells, phospho-ERK directly interacts with OCCLUDIN and prevents H_2_O_2_-induced disruption of TJs and increased paracellular permeability^48^. Furthermore, in a total-parenteral-nutrition (TPN) rat model, the p38 MAPK inhibitor SB203508 significantly suppressed TPN-mediated intestinal permeability^51^. In Caco-2 cells, SB203580 treatment also inhibited IL-1β-induced increase in tight junction permeability via repressing the p38/ATF-2 signalling^51^. Our study particularly highlighted an essential role of JNK MAPK isoform in the modulation of barrier function by plasma obtained from CD patients and provides new evidence for potential applications of JNK inhibition in IBD therapy.

In the present study, we used a well-established 3D Caco-2 cyst model^52^. Compared to the widely used 2D intestinal cell monolayers, 3D cyst with a single lumen recaptures typical morphological and biological features of intestinal epithelial *in vivo*, such as expression of brush border enzymes and formation of microvilli^53^. Moreover, Caco-2 monolayers are shown to have tighter cell junctions and to be more resistant to stressors than the human intestinal epithelium^54,55^. As a comparison, we also exposed 2D Caco-2 monolayers to TNF-α and observed that only basollateral TNF-α concentration greater than 1 ng/mL were able to impair the paracellular barrier (Supplementary Fig. 3a), while treatment with a concentration of 25 pg/mL, in the range of those found in blood of CD patients, to the 3D Caco-2 cysts already resulted in a significant increase in the paracellular permeability (Fig. 3a,b). These data demonstrated that 3D Caco-2 culture system allows us to study the effect of stimuli at much lower and physiological concentrations, and serves as an excellent model system to study intestinal barrier function and putative mechanisms. Moreover, in our study, Caco-2 cysts were exposed to plasma at the basal side, which mimics *in vivo* basolateral exposure of the intestinal epithelium and provides insights into the role of systemic compartment in the modulation of barrier function. However, in future studies, it would also be interesting to assess effects of different stimuli (patient plasma, intestinal lumen contents, pro- or anti-inflammatory mediators, and IBD-related drugs) on barrier function through exposure at the basolateral or luminal side of the 3D Caco-2 model.

Noteworthy, we have included plasma samples from only 6 subjects per group in the current study. Although we could demonstrate that plasma from all the CD patients with various disease phenotypes had an increased disrupting effect on barrier function as compared to healthy controls, and the impairing impact correlates with disease activity, the small sizes of the subgroups precluded us from performing additional analyses to investigate whether other factors such as drug use or disease location/behaviour were related to plasma-modulated intestinal permeability. Moreover, in this study disease activity was defined based on the Harvey-Bradshaw Index (HBI). Although we are aware of the rather poor correlation with mucosal inflammation, we think that our current findings still point to potential differences in barrier function in relation to disease activity. Further studies including a larger number of well-phenotyped patients with longitudinal follow-up, including endoscopic and/or fecal calprotectin scores are warranted to further unravel the contribution of barrier dysfunction in the development of exacerbations.

In summary, our study showed for the first time that plasma from CD patients is able to induce epithelial barrier dysfunction, partly by TNF-α induced TJs modulation, with a pronounced involvement of JNK MAPK signalling pathway. Moreover, we revealed that precondition of adalimumab and SP600125 could inhibit the damaging effect of plasma from CD patients on epithelial barrier function. These findings highlight the relevance of the systemic compartment as a contributing factor in promoting disease development, and underscore intestinal barrier restoration as a potential mechanism that contributes to the clinical benefits of adalimumab and JNK inhibitor in CD patients. In conclusion, our findings provide new insights into understanding the regulation of barrier function and its role in the pathogenesis of IBD. We also provide new basic evidence that combined treatment with JNK inhibitor and TNF blocker might be beneficial in IBD therapy.

## Methods

### Study population and sample

This study is part of the population-based IBD South Limburg (IBDSL) cohort study^56^, which has been approved by Maastricht University Medical Centre + Committee of Ethics (NL31636.068.10). The study was executed according to the revised version of the Declaration of Helsinki (Seoul, South Korea, Oct. 2008) and all subjects had signed an informed consent prior to participation. Patients with active CD (n = 6) and inactive CD (n = 6) and healthy controls (HC, n = 6) were recruited in the present study (Supplementary Table 1). Clinical disease activity was graded according to the well-accepted Harvey-Bradshaw Index (HBI) with validated cut offs to define active disease and remission. Active disease was defined as an HBI score of more than 4^57^. Phenotype data according to the Montreal classification^58^ and medication use were retrieved from the IBDSL data warehouse^56^. From each subject, blood samples were collected in pre-cooled Sodium-Heparin tubes and stored on ice. Within 2 hours, samples were centrifuged (4000 rpm; 4 °C; 10 min), supernatant was collected, aliquoted and stored at −80 °C until further experiments.

### Three-dimensional cell culture

Caco-2 cells (ATCC, Rockville, USA) were maintained in Dulbecco’s Modified Eagle Medium (DMEM; Lonza Benelux BV, Breda, the Netherlands) containing 4.5 g/l glucose and L-glutamine, 10% (v/v) fetal calf serum (FBS, Invitrogen, Breda, the Netherlands), 1% (v/v) solution of non-essential amino acids (NEAA, Invitrogen) and 1% (v/v) solution of antibiotic/antimycotic mixture (anti-anti, Invitrogen, Breda, the Netherlands) in an atmosphere of 5% CO_2_ at 37 °C. Caco-2 cells were grown in growth factor-reduced Matrigel^®^ (8 mg/ml; BD Biosciences, San Jose, California USA) in 10 mm glass bottom culture dishes of 35 mm diameter (MatTek Corporation, Ashland, USA) for barrier function analyses, in 4-well µ-dishes (Ibidi GmbH, Planegg/Martinsried, Germany) for immunofluorescent staining, and in 96-well plates (Corning BV, Amsterdam, the Netherlands) for cell viability analysis^30^. Briefly, Caco-2 (2.5 × 10^4^ cells/ dish; passage 29–46) were embedded in 40% growth factor-reduced Matrigel® and solidified at 37 °C for 30 min in culture dishes. Thereafter, medium (DMEM with 10% FBS, 1% NEAA and 1% anti-anti) was added and cysts were allowed to grow for 5–7 days at 37 °C^30^. The quality of cultures was checked by counting the number of high quality cysts and classifying them according to the number of lumens formed. Only cultures containing >70% of cysts with a single lumen were used for further experiments.

### Exposure of human plasma, adalimumab, TNF-α and SP600125

Caco-2 cysts were incubated basolaterally with either 2 ml solution containing 37.5% (v/v) plasma from active or inactive CD patients and healthy controls (HC) for a period of 24 hours at 37 °C, or with 25 pg/mL TNF-α (Cell Signalling Technology, Danvers, MA) for 24 hours at 37 °C. In all treatments, cysts were pre-incubated with or without 10 or 20 μg/mL of the TNF-blocker adalimumab (HUMIRA, Abbott Laboratories, Wiesbaden, Germany), or 100 nmol/L SP600125 (Sigma-Aldrich, Amsterdam, The Netherlands). Medium only and 2 mM ethylene glycol tetraacetic acid (EGTA) were used to serve as negative and positive controls, respectively.

### Determination of paracellular barrier function

To assess barrier function, cysts were incubated with 10% (v/v) fluorescein isothothiocyanate-labelled dextran of 4 kDa (FITC-D4; Sigma-Aldrich, Amsterdam, The Netherlands) for a period of 24 hours at 37 °C. Paracellular permeability was determined by the flux of FITC-D4 from the basal to the luminal compartment using confocal microscopy, and quantified as luminal/basal (L/BL) ratio. Confocal images were taken with Leica TCS SPE confocal laser scanning microscope (Leica Microsystems GmbH, Mannheim, Germany) and processed using TCS SPE browser and Image J software^59^.

### Transepithelial electrical resistance measurements (TEER)

Caco-2 monolayers at the density of 1 × 10^5^/well were seeded on collagen-coated permeable polycarbonate Transwell filters (Costar, Cambridge, MA, USA) and cultured for 3 weeks. Before and after various basolateral treatments (including plasma, TNF-α, adalimumab or SP600125 at the above described concentrations), TEER values were measured using EVOM Epithelial Voltohmmeter (World Precision Instruments, Berlin, Germany) and chopstick-style electrodes. TEER of all inserted wells were normalized to the corresponding 0 h value and are presented as percentage of initial value.

### Cell viability assessment

Cell viability was determined by measuring the release of lactate dehydrogenase (LDH) using the LDH essay (CytoTox ONE^tm^; Promega, the Netherlands) according to manufacturer’s instructions^30^. Briefly, Caco-2 cysts in 96-well plates were incubated with plasma from active and inactive CD patients and HC. Plates were incubated at 37 °C for 24 hours and equilibrated at room temperature (RT) for 20 min. Then 100 µL of the substrate mix was added and incubated at RT, protected from light for another 30 min, followed by addition of 20 µL stop solution. Maximum LDH release was induced by using lysis solution. The fluorescence was measured at an excitation and an emission wavelength of 560 nm and 590 nm, respectively. The percentage of LDH activity was calculated as percentage of maximum LDH release from fully lysed cells.

### Immunofluorescence analyses of ZO-1 and OCCLUDIN

Caco-2 cysts were fixed in 4% (w/v) paraformaldehyde in PBS at RT for 40 min and processed for immunocytochemistry as described previously with minor modifications^52^. Briefly, cysts were permeabilized with 0.5% (v/v) Triton X-100 in PBS at RT for 30 min and incubated with 1% (w/v) BSA for 1 hour. Cysts were then incubated at 4 °C overnight with conjugated mouse anti-ZO-1 (Life Technologies Europe BV, Bleiswijk, Netherlands) and/or rabbit anti-OCCLUDIN (Life Technologies, Bleiswijk, Netherlands) at 1:200 dilution, then washed three times with PBS for 5 min, and then stained for 10 min at RT with diamidino-2-phenylindole (DAPI; Sigma Chemical Co) at 1:1500 dilution. After two washings with PBS, cysts were mounted in dishes using VectaShield mounting medium (Vector Laboratories, Burlingame, USA). Confocal images were obtained using a Leica TCS SPE confocal laser scanning microscope (Leica Microsystems GmbH) with identical acquisition settings (laser power, objectives, magnifications) for each acquired image and condition. Fluorescence quantification of the confocal images was done using Image J software^59^. Cysts contacting other cysts or located at the image border were excluded for quantitative analyses. 5 cross-section views per biological replicate were collected. For each cross-section image, the total surface (covering ZO-1, OCCLUDIN or DAPI staining signals) was marked as the region of interest by manually defining the out border of the cyst. A threshold was applied to refine the detection. The total pixel intensities of ZO-1, OCCLUDIN or DAPI were subtracted with the background pixel intensity. The fluorescence intensities of ZO-1 and Occludin were then normalized to that of DAPI from the same cross-section.

### RNA isolation and real time PCR

Caco-2 cells (50 × 10^3^ cells/well) were cultured in 3D in 12-well plates (Corning BV, Amsterdam, the Netherlands). After treatment, RNA was extracted using Qiagen RNeasy Mini kit (QIAGEN, Germantown, USA), according to manufacturer’s instructions. Briefly, 3D cysts were lysed and placed on a rotating platform (100 rpm) for 10 min at RT. Next, the lysate was homogenized and transferred to an RNeasy spin column and centrifuged for 15 seconds at 8.000 × g. Thereafter, RNA was eluted in 50 µl RNase-free water and its concentration was measured using NanoDrop (NanoDrop Products, Wilmington, USA). Gene expression of *ZO-1*, *OCCLUDIN, MARVELD2, LSR, ILDR-1 and ILDR-2* was evaluated by qPCR as described previously^52^. Primer sequences are given in Supplementary Table 2.

### ELISA assessment of ERK1/2, p38 and JNK phosphorylation

Caco-2 3D cysts in 96-well plates were exposed to plasma from active, inactive CD patients or HC for 24 h at 37 °C and 5% CO_2_. Thereafter, cysts were processed for cell-based ELISA and incubated with rabbit anti-human antibodies (Cell Signalling Technology, Leiden, The Netherlands) against p-p38 (#9258P4511P), p38 (#9212P), p-ERK1/2 (#4370), ERK1/2 (#4695), p-JNK (#4668) and JNK (#9252) with 1:100 dilution in the blocking solution Ray Biotech), followed by HRP-conjugated anti-rabbit IgG (Dako, Glostrup, Denmark) as we described previously^60^. Finally, 3, 3′, 5, 5′-Tetramethylbenzidine (TMB) was added, followed by stop solution (2N H_2_SO_4_) and optical density was read at 450 nm by SpectraMax M2 spectrophotometer (Molecular Devices).

### Western blotting analyses

3D Caco-2 cysts were lysed with RIPA buffer containing 1% protease inhibitor and 1% phosphatase inhibitor cocktails (Sigma-Aldrich, Amsterdam, NL). Protein concentration was equalized then subjected to 12% SDS-PAGE separation, and then transferred to PVDF membrane (GE healthcare, Amsterdam, NL). After blocking for 1 hour with 5% milk at RT, the membranes were then incubated overnight at 4 °C with primary rabbit anti-human antibodies (Cell Signalling Technology, Leiden, NL) against p-p38, p38, p-ERK1/2, ERK1/2, p-JNK and JNK with 1:1000 dilution, and then against horseradish peroxidase-conjugated goat anti-rabbit IgG (Cell Signalling Technology, Leiden, NL) with 1:4000 dilution. After final washes, membranes were incubated in ECL western blotting detection reagent, and detection was performed using Gel Doc XR + Imaging system (Bio-Rad).

### Statistical analyses

Descriptive data of the subjects were analysed using SPSS (IBM SPSS Statistics for Windows, version 19.0). All experiments were performed three times in triplicate and results were reported as means ± SEM of at least 8 cysts per experiment. Data were analysed parametrically. One-way analysis of variance (ANOVA) and Tukey’s post-hoc test was performed to determine significant differences between different treatment conditions using GraphPad (GraphPad Software Incorporated, version 6.0). *P* < 0.05 was considered significant.

## Supplementary information

Supplementary Information
