# Supplementary material for: Modulation of Intestinal Epithelial Permeability by Plasma from Patients with Crohn’s Disease in a Three-dimensional Cell Culture Model
Source: Sci Rep. 2019 Feb 14;9:2030. doi: 10.1038/s41598-018-38322-8 (PMC6375954; doi:10.1038/s41598-018-38322-8)
Supplement: Supplementary file 1 — Supplementary Information [file 41598_2018_38322_MOESM1_ESM.pdf]

Supplementary Information

Modulation of Intestinal Epithelial Permeability by Plasma from Patients with Crohn's Disease in a Three-dimensional Cell Culture Model

Pan Xu [1, 2], Elhaseen Elamin [1, 2], Montserrat Elizalde [1, 2], Paul Bours [1], Marieke Pierik [1, 2], Ad Masclee [1, 2], Daisy Jonkers [1, 2*]

Affiliation: [1]Division of Gastroenterology-Hepatology, Department of Internal Medicine, Maastricht University Medical Centre, Maastricht, the Netherlands, [2]School for Nutrition, Toxicology and Metabolism of Maastricht University Medical centre, the Netherlands.

* Authorship note: Pan Xu and Elhaseen Elamin contributed equally to this work.

Corresponding author:

Daisy Jonkers PhD

Department of Internal Medicine, Division of Gastroenterology and Hepatology, Maastricht University Medical Centre, P. Debyelaan 25, 6229 HX Maastricht, The Netherlands.

Tel: +31-043-3884266, Fax: +31-43-3874692.

Email: d.jonkers@maastrichtuniversity.nl

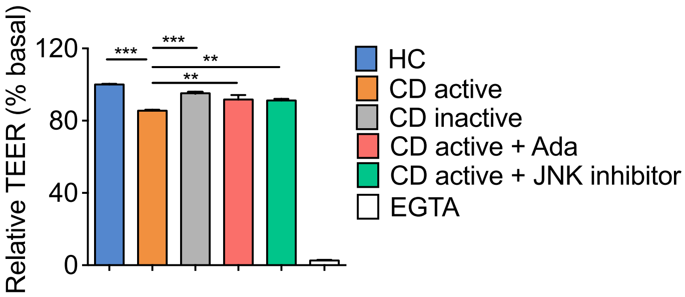

Supplementary Figure 1. Effects of plasma from patients with Crohn's disease on transepithelial electrical resistance (TEER) in Caco-2 monolayers, with or without the pre-incubation of adalimumab (20 µg/mL) or JNK inhibitor SP600125 (100 nmol/L). 2D Caco-2 monolayers were treated at the basolateral side with plasma (37.5% v/v) from healthy controls (HC), active or inactive CD patients for 24 hours. EGTA (2 mM) was used as positive control. Bars are expressed as means ± SEM of three independent experiments. (*) $P<0.05$; (**) $P<0.01$ by one-way ANOVA and Tukey's post-hoc test.

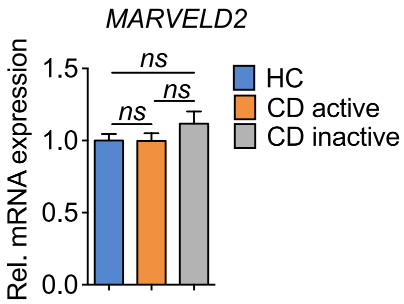

Supplementary Figure 2. Relative mRNA levels of *MARVELD2* in Caco-2 cysts that were treated with plasma (37.5% v/v) from healthy controls (HC), active or inactive CD patients for 24 hours. Data expressed as means ± SEM with 6 subjects per group and at least 8 cysts per subject. Statistical analyses were done by one-way ANOVA and Tukey's post-hoc test.

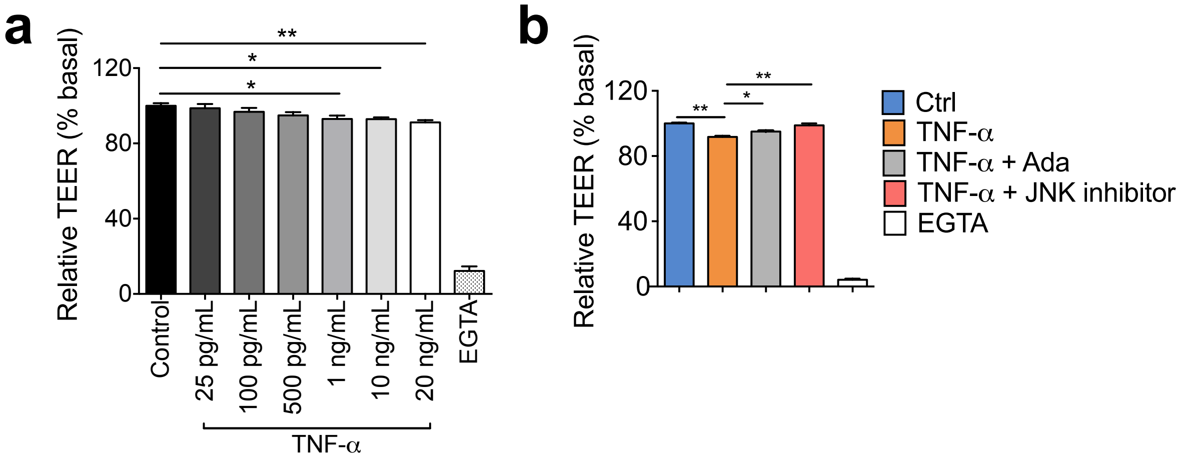

Supplementary Figure 3. Effects of TNF-α on transepithelial electrical resistance (TEER) in Caco-2 monolayers, with or without the pre-incubation of adalimumab (20 μg/mL) or JNK inhibitor SP600125 (100 nmol/L). TNF-α at different concentrations (**a**) and at concentration of 10 ng/mL (**b**) were added to the basolateral side of Caco-2 cells for 24 hours. EGTA (2 mM) was used as positive control. Bars are expressed as means ± SEM of three independent experiments. (*) P<0.05; (**) P<0.01 by one-way ANOVA and Tukey's post-hoc test.

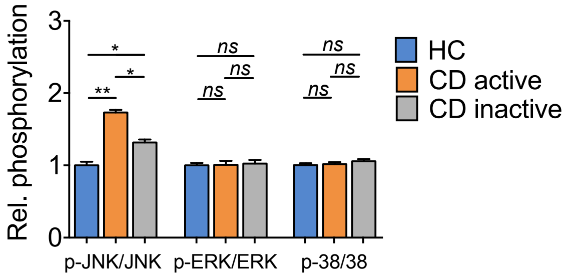

Supplementary Figure 4. Quantitative densitometry of western blot (Fig. 5a) on phosphorylation levels of ERK1/2, p38 and JNK in Caco-2 cysts that were exposed to plasma (37,5%) from healthy control (HC), active or inactive CD patients. Data are expressed as means ± SEM of three independent experiments. (*) $P<0.05$; (**) $P<0.01$ by one-way ANOVA and Tukey's post-hoc test.

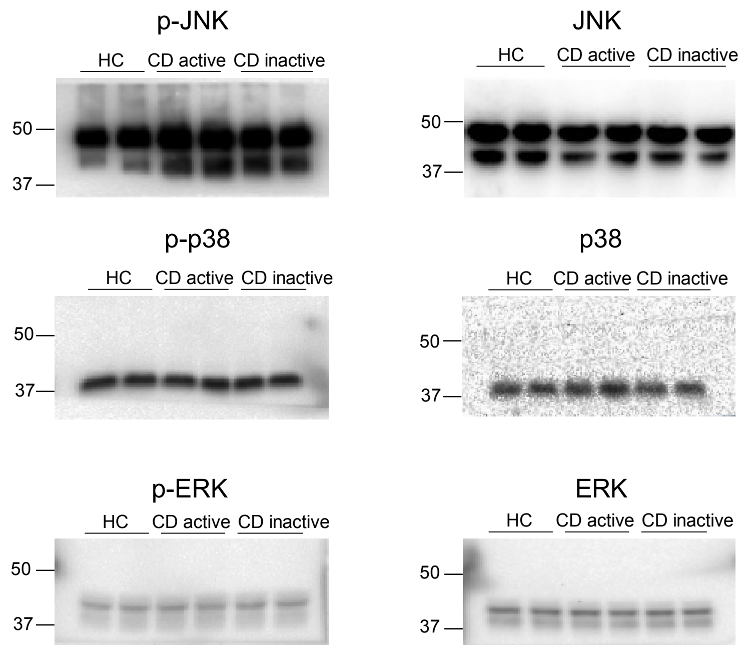

Supplementary Figure 5. Full size western blot images as shown in Fig. 5a.

Supplementary Table 1: Baseline characteristics of study population

	HC (n = 6)	Active CD (n = 6)	Inactive CD (n = 6)
Age (year, mean ± SD)	35,2 ± 14,8	37,2 ± 19,4	40,2 ± 12,2
Sex (male/female)	2/4	1/5	3/3
Disease duration (year, mean)	-	8,7 ± 10,4	10 ± 8,2
HBI (mean ± SD)	-	7,2 ± 3,5	1 ± 1,1
Age at diagnosis[1] (n)			
A1	-	1	1
A2	-	4	3
A3	-	1	2
Location of the disease[1] (n)			
L1	-	2	2
L2	-	2	2
L3	-	2	2
Disease behaviour[1](n)			
B1	-	4	5
B2	-	0	1
B3	-	2	0
Medication*			
Mesalazine	-	0	0
Prednisone	-	2	0
Anti-TNF	-	3**	2***
Azathioprine	-	1	3
Methotrexate	-	2	0

1 According to the Montreal classification. L1 corresponds to disease in the terminal ileum, L2 to disease in the colon, L3 to disease in the ileocolon, and L4 to disease in the isolated upper gastrointestinal tract. B1 corresponds to non-stricturing, non-penetrating, B2 to stricturing, and B3 to penetrating.

\- Not available

HBI Harvey-Bradshaw Index

* Total is higher than the number of patients as some of them receive combination therapy

** 2 patients receive adalimumab, 1 patient receives infliximab

*** 2 patients receive infliximab

Supplementary Table 2: Primer sequences for qPCR

Gene	Forward primer	Reverse primer
18S	GTAACCCGTTGAACCCCATT	CCATCCAATCGGTAGTAGCG
ZO-1	AGGGGCAGTGGTGGTTTTCTGTTCTTTC	GCAGAGGTCAAAGTTCAAGGCTCAAGAGG
OCCLUDIN	TCAGGGAATATCCACCTATCACTTCAG	CATCAGCAGCAGCATGTACTCTTCAC
MARVELD2	TCAGACAGATGATGAGCGAGA	ATGTTCCTGTCGGCTTTCC
LSR	CAACAGGACGGACTTGGAGTA	AGAAGCCACACGAAGACGAC
ILDR-1	TCCTTGCTTGTGACGGTCC	CAAAGATAGGGTCCTTGCAGAAG
ILDR-2	AGTGCCCGACAAGAAGAAGG	CGATCCTGGCAGTAGGACT
